# Edoxaban, a Factor Xa-Specific Direct Oral Anticoagulant, Significantly Suppresses Tumor Growth in Colorectal Cancer Colon26-Inoculated BALB/c Mice

**DOI:** 10.1055/s-0042-1758855

**Published:** 2023-01-07

**Authors:** Keiichi Hiramoto, Nobuyuki Akita, Junji Nishioka, Koji Suzuki

**Affiliations:** 1 Department of Molecular Pathobiology, Faculty of Pharmaceutical Sciences, Suzuka University of Medical Science, Suzuka-city, Mie, Japan; 2 Department of Clinical Engineering, Faculty of Medical Engineering, Suzuka University of Medical Science, Suzuka-city, Mie, Japan; 3 Department of Clinical Nutrition, Faculty of Health Science, Suzuka University of Medical Science, Suzuka-city, Mie, Japan

**Keywords:** DOAC, edoxaban, PAR2, Colon26 cells, tumor growth, BALB/c mice

## Abstract

**Introduction** Certain low-molecular-weight heparins have been reported to reduce tumor growth and metastasis in tumor cell-inoculated mouse models and cancer patients. Recently, direct oral anticoagulants (DOACs) have been widely used in patients with thromboembolism. This study was aimed at investigating the effect of DOACs, which target thrombin or factor Xa, on tumor growth in a syngeneic mouse model comprising BALB/c mice inoculated with colon cancer Colon26 cells.

**Materials and Methods** DOACs targeting thrombin (dabigatran etexilate [DABE]) or factor Xa (rivaroxaban [RVX] and edoxaban [EDX]) were orally administered daily to male BALB/c mice inoculated with Colon26 cells, followed by analyses of tumor growth and plasma levels of coagulation- and tumor-related factors such as tissue factor (TF), plasminogen activator inhibitor-1 (PAI-1), interleukin-6 (IL-6), and matrix metalloproteinase-2 (MMP-2).

**Results** Colon26 cells expressed significant amounts of functionally active TF. Tumor growth in Colon26-inoculated mice was significantly suppressed in DABE- or RVX-treated mice ( *p* <0.05) and was suppressed more significantly in EDX-treated mice ( *p* <0.01). Therefore, the antitumor mechanism of action of EDX was investigated next. Plasma levels of TF, PAI-1, IL-6, and MMP-2 were elevated in Colon26-inoculated mice but were significantly reduced in EDX-treated mice ( *p* <0.01). The expression of protease-activated receptor (PAR)1, PAR2, signal transducer and activator of transcription-3 (STAT3), cyclin D1, and Ki67 was increased in tumor tissue of Colon26-inoculated mice but (except for PAR1) was significantly decreased in tumor tissues of EDX-treated mice ( *p* <0.01). In addition, apoptotic cells and p53 protein levels were significantly increased in tumor tissues of EDX-treated mice.

**Conclusion** The data suggest that among the tested DOACs, EDX significantly suppresses tumor cell proliferation via the factor Xa-PAR2 pathway, which is activated by coagulation and inflammation in Colon26-inoculated mice and induces tumor cell apoptosis.

## Introduction

Patients with leukemia, pancreatic cancer, and lung cancer are more likely to develop deep vein thrombosis or cerebral thromboembolism (Trousseau syndrome), which leads to stroke in approximately 25% of cases. 1 Tumor cells activate the hemostatic system in multiple ways and may release microparticles containing the procoagulant tissue factor (TF) and cancer procoagulants that can directly activate the coagulation cascade. Tumor cells may also activate the host's hemostatic cells (endothelial cells and platelets) by either releasing soluble factors or by direct adhesive contact, thus further enhancing clotting activation. 2 Traditionally, anticoagulant therapy with low-molecular weight heparin (LMWH) or warfarin has been used to treat such thromboembolism. 3 Recently, direct oral anticoagulants (DOACs) have been widely used in place of warfarin to prevent the onset of cerebral thromboembolism in patients with non-valvular atrial fibrillation and thromboembolism after surgery on the knee and hip joints of the lower extremities. 4 5 6 7

Anticoagulation therapy may reportedly reduce tumor size in cancer patients with thrombosis; LMWHs (specific for factor Xa), such as dalteparin 8 and nadroparin, 9 significantly improve survival in patients with cancer compared with treatment without anticoagulant therapy. In preclinical studies, tinzaparin and other LMWHs interfered with cancer-induced hypercoagulation, cancer cell proliferation, degradation of the extracellular matrix, angiogenesis, chemokine signaling, tumor progression, and metastasis. 10 However, subsequent studies and meta-analyses showed no effect of LMWH on the survival of cancer patients. 11 The current consensus is that LMWH does not improve the survival of patients with cancer, although there are several positive results in some studies. 12 13

Recently, it was reported that pre-treatment with anticoagulant and anti-inflammatory drugs, such as human activated protein C 14 and recombinant soluble human thrombomodulin (rTM), 15 which is used for treating disseminated intravascular coagulation in Japan, significantly inhibited metastasis of B16 melanoma (B16) cells transplanted into C57BL/6j mice transgenic for human protein C inhibitor. 16 rTM also suppressed the growth of CT26. CL25 cells transplanted into BALB/c mice. 17 These data suggest that anticoagulants have tumor-suppressive activity in mice.

This study shows the effect of DOACs (thrombin inhibitor: dabigatran etexilate [DABE] and factor Xa inhibitor: rivaroxaban [RVX] and edoxaban [EDX]) on tumor growth in mice inoculated with mouse-derived colorectal cancer Colon26 cells. 18 Next, based on the finding that EDX significantly inhibited tumor growth in Colon26-inoculated mice, the basic mechanism of tumor growth inhibition by EDX in mice inoculated with Colon26 cells was analyzed.

## Materials and Methods

### DOACs

The thrombin inhibitor, prodrug DABE, was purchased from Nippon Boehringer Ingelheim Co., Tokyo, Japan, the factor Xa inhibitor, RVX, from Bayer Holding, Tokyo, Japan, and EDX was from Daiichi-Sankyo Co., Tokyo, Japan. Each DOAC reagent was dissolved in dimethyl sulfoxide and diluted with saline at different concentrations before being orally administered to mice: DABE (250 mg/mL), RVX (25 mg/mL), or EDX (50 mg/mL), and stored at −30°C until use.

### Measurement of mRNA of Coagulation and Inflammation Factor Genes in Colon26 Cells

Colon26 cells 18 produced as colorectal cancer by a single rectal administration of *N* -nitroso-N-methyl urethane to BALB/c mice are available at the Cell Resource Center for Biomedical Research, Institute of Development, Aging, and Cancer, Tohoku University. To measure the mRNA expression of coagulation and inflammation factor genes in Colon26 cells, cultured cells were lysed in a cell lysis preservation solution (Buffer RLT; Qiagen, Hilden, Germany) and frozen until use. Total RNA extracted from the cells was used to prepare cDNA using an Omniscript Reverse Transcription Kit (Qiagen). The mRNA expression levels of mouse genes encoding TF, factor VII, factor X, plasminogen activator inhibitor-1 (PAI-1), PAR1, PAR2, and interleukin-6 (IL-6) were determined using polymerase chain reaction (PCR) with the primers listed in Table 1 . The protocol for PCR was as follows: (35 cycles) denaturation at 94°C for 30 seconds, annealing at 59°C for 30 seconds, and extension at 72°C for 60 seconds. The amplified products were separated by agarose gel electrophoresis and semi-quantitatively analyzed using a ChemiDoc Imaging System (Bio-Rad Laboratories, California, United States).

**Table 1:** Primers used for the semi-quantification of mRNA levels of coagulation and inflammatory factors in cultured Colon26 cells

	Forward primer	Reverse primer
*TF*	5′-CAAGTGCTTCTCGACCACAGA-3′	5′-GTGCACACTGTACTGCTTCCTG-3′
*FVII*	5′-TGTGCCTCGAATCCATGTCA-3′	5′-CGGTCACTATCCATCTGGCG-3′
*FX*	5′-TCTTGCATCTCCACAGCTCC-3′	5′-CCTCAGCACGGCGATATCAT-3′
*Pai1*	5′-CGTCTCTGTGCCCATGATG-3′	5′-TCGTTTACCTCGATCCTGACC-3′
*Par1*	5′-TGAGCCAGCCAGAATCAGAG-3′	5′-ACCGACACGAAGAGCACGT-3′
*Par2*	5′-GCTCCGAGTTTCGAACCACT-3′	5′-AAGCCAATGAGCACCTTGC-3′
*l16*	5′-GATACCACTCCCAACAGACCTG-3′	5′-CACTCCTTCTGTGACTCCAGCT-3′
*GAPDH*	5′-ATGCCCCCATGTTTGTGAT-3′	5′-CCTGCTTCACCACCTTCTTG-3′

Abbreviations: FVII, factor VII; FX, factor X; GAPDH, glyceraldehyde 3-phosphate dehydrogenase; Il-6, interleukin-6; Pai1, plasminogen activator inhibitor-1; Par1, protease-activated receptor-1; Par2, protease-activated receptor-2; TF, tissue factor.

### Western Blot Analysis of TF in Colon26 Cells

Cultured Colon26 cells (10 ^6^ cells) were homogenized with a lysis buffer containing 0.5% Nonidet P-40, 10% glycerol, 137 mM NaCl, 2 mM ethylenediaminetetraacetic acid, and 50 mM Tris-HCl buffer (pH 8.0). After centrifugation at 8,000 × *g* for 10 minutes, the supernatant fraction was collected, and the total protein concentration of the cell extract was measured using Bradford Protein Assay Kit (Bio-Rad Laboratories, Hercules, California, United States) and stored at −80°C until analysis. Samples (0.25–2.5 μg total protein/lane) were subjected to 10% polyacrylamide gel electrophoresis in the presence of 0.1% sodium dodecyl sulfate. The electrophoresed protein was transferred to an Immobilon membrane (Life Technologies, Carlsbad, California, United States), subsequently blocked with 5% skim milk at 4°C overnight, and incubated with anti-mouse TF monoclonal antibody (1:1,000; Bioss Antibodies Inc., Woburn, Massachusetts, United States) at 25°C for 1 hour. The membrane was then treated with a horseradish peroxidase-conjugated secondary antibody (Novex, Frederick, Maryland, United States). Immune complexes were detected using ImmunoStar Zeta regent (Wako, Osaka, Japan), and images were acquired using the Multi-Gauge software program (Fujifilm, Greenwood, South Carolina, United States).

### Measurement of TF Activity in Colon26 Cells

The TF activity of Colon26 cells was determined by measuring the duration of tissue thromboplastin-induced plasma clotting in the presence of Ca ^2+^ ions using a coagulation time measuring instrument CA-600 (Sysmex, Kobe, Japan). Briefly, to a cuvette incubated at 37°C, 50 µL of citrated normal human plasma (Sysmex) was added, and after 1 minute, 50 µL of a suspension containing various numbers of Colon26 cells was added, and then 50 µL of 20 mM CaCl _2_ solution was added to measure plasma clotting time. To generate a calibration curve for TF activity measurement, 50 µL of a commercially available tissue thromboplastin reagent, Thromborel S (Siemens Health Care Diagnostic, Marburg, Germany), diluted with Tris-buffered saline (TBS, pH 7.5) from 1/10 to 1/1,000, was used instead of a suspension of Colon26 cells.

### Evaluation of Antitumor Effects of DOACs in Mice

To assess the antitumor effects of DOACs, BALB/c mice inoculated with Colon26 cells were used. The method of transplanting Colon26 cells into BALB/c mice constitutes a syngeneic model for observing cancer tissue growth that does not require immunosuppressive animals and is more suitable for studies of antitumor activity. The oral dose of DOAC was determined according to that previously used in preclinical studies using mice for the development of DABE, 19 RVX, 20 and EDX. 21 In the first experiment, white male BALB/c mice (6 weeks old) were inoculated subcutaneously with Colon26 cells (10 ^5^ cells/200 µL/mouse) in the thigh; on day 7 (when cell engraftment was expected), the mice were divided into four groups and treated as follows: Tap water (control: 200 μL water/mouse; *n* = 5), DABE (50 mg/kg/200 μL/mouse; *n* = 5), RVX (5 mg/kg/200 μL/mouse; *n* = 5), or EDX (10 mg/kg/200 μL/mouse; *n* = 5) was administered daily via feeding needle until day 21. The dose of each DOAC is a concentration that does not cause bleeding but significantly prolongs the coagulation time and exhibits an antithrombotic effect in mice. 19 20 21 To prevent the development of thromboembolism in patients, DABE is commonly administered orally at 3.5 to 5.0 mg/kg/d, RVX at 0.2 to 0.3 mg/kg/d, and EDX at 0.5 to 1.0 mg/kg/d. Therefore, in this experiment, the dose of DOAC in mice was approximately 10 times the dose in humans per body weight, as previously used in preclinical studies using mice. 19 20 21

In the next experiment, dose–response of EDX (0, 5, 10, and 20 mg/kg/200 μL/mouse; *n* = 5) was evaluated. Mouse body weights and tumor volumes were measured weekly during feeding. The major and minor axes of tumors were measured using a caliper, and the tumor volume was calculated as follows: major axis × major axis × minor axis × ½. On day 21, mice were anesthetized, normal or tumor tissue was taken from the thigh, and blood was taken from the heart. Blood was mixed with 1/10 volume of 100 IU/mL sodium heparin and centrifuged at 3,000 × *g* at 4°C for 10 minutes using a desktop cooling centrifuge. The tissues and heparinized plasma were stored at −80°C for later use.

### Histological Analysis of Normal and Tumor Tissues

Tumor tissue of the thigh subcutaneously inoculated with Colon26 cells and normal thigh tissue of untreated (UTR) mice was used for histological analysis after hematoxylin-eosin (HE) staining. The tissue sections included the skin dermis, subcutaneous tissue, muscularis mucosae, and the presence or absence of tumor tissue.

To visualize the tissue cells expressing protease-activated receptors (PAR) for thrombin (PAR1) or factor Xa (PAR2), the cell growth factor signal transducer and activator of transcription-3 (STAT3), the cell cycle regulator cyclin D1, and Ki67, the tissue fragments were treated with the respective primary antibodies: anti-PAR1 (Abbiotec, San Diego, California, United States), PAR2 (Santa Cruz Biotechnology, Santa Cruz, California, United States), anti-STAT3 (Cell Signaling Technology, Danvers, Massachusetts, United States), anti-cyclin D1 (Bioss, Woburn, Massachusetts, United States), and anti-Ki67 (Santa Cruz Biotechnology), followed by treatment with a fluorescent dye-conjugated secondary antibody (1:30; Dako Cytomation, Glostrup, Denmark) and observed under a fluorescence microscope (Olympus, Tokyo, Japan).

### Evaluation of Apoptotic Cells in Normal and Tumor Tissues

Apoptotic cells in the normal tissue and tumor tissue sections were evaluated using an apoptosis detection kit (Takara Biomedicals, Tokyo, Japan) and expressed as the number of terminal deoxynucleotidyl transferase dUTP nick end labeling (TUNEL)-positive cells. 22 The number of TUNEL-positive cells in five visual fields was counted under a fluorescence microscope using Image J software. Tissues were treated with 4',6-diamidino-2-phenylindole (DAPI) stain (Thermo Fisher Scientific; 0.5 μg/mL in TBS) for 3 minutes and washed three times with 2 mL TBS and dried, following which a non-fluorescent encapsulant was added (Fluoromount Aqueous Mounting Medium; Empire Genomics LLC, Buffalo, New York, United States).

### Determination of Coagulation- and Tumor-related Factors in Plasma and Tissues

Tumor tissue samples were collected on the last experimental day. Normal tissues of the lower limbs and thighs from untreated mice or tumor tissues (100 mg) were rinsed in phosphate-buffered saline (PBS) to remove excess blood before homogenization. The tissues were finely minced and homogenized in 1 mL of PBS with a glass homogenizer on ice. The concentrations of IL-6, matrix metalloproteinase-2 (MMP-2), TF, and PAI-1 in heparinized plasma and those of PAR1, PAR2, STAT3, cyclin D1, Ki67, p53, VEGF-A, and VEGFR-1 in normal or tumor tissues were determined by enzyme-linked immunosorbent assay (ELISA) using *mouse IL-6 quantikine ELISA kit (M6000B; R&D Systems, Minneapolis, Minnesota, United States),* mouse MMP-2 ELISA kit (ab254516; Abcam, Cambridge, UK), mouse TF ELISA kit (SEA524Ra; Cloud-Clone, Houston, Texas, United States), mouse PAI-1 ELISA kit (ab197752; Abcam), mouse PAR1 ELISA kit (MBS753326; MyBioSource, San Diego, California, United States), mouse PAR2 ELISA kit (MBS4501658; MyBioSource), mouse STAT3 ELISA kit (OKEH02995; Aviva System Biology, San Diego, California, United States), cyclin D1 ELISA kit (NOV596744; Novus, Centennial, California, United States), mouse Ki67 ELISA kit (EM1473; Wuhan Fine Biotech, Hubei, China), mouse p53 ELISA kit (ab224878; Abcam), mouse VEGF-A ELISA kit (BMS619–2; Thermo Fisher Scientific, Waltham, Massachusetts, United States), and mouse VEGFR-1 ELISA kit (CSB-E04762m; CUSABIO, Houston, Texas, United States) respectively, according to the manufacturer's instructions.

### Evaluation of Coagulation Ability in Plasma from Colon26-Inoculated Mice

To evaluate the effect of oral treatment with EDX on coagulation ability in plasma from Colon26-inoculated mice, changes in the levels of the coagulation marker thrombin-antithrombin (TAT) complex in plasma were determined.

Briefly, white male BALB/c mice (6 weeks old) were subcutaneously inoculated with Colon26 cells (10 ^5^ cells/200 µL/mouse) into the lower thigh. After 7 days (day 0), the mice were divided into two groups and treated daily as follows: oral treatment via feeding needle with tap water (200 μL water/mouse; *n* = 5) or EDX (10 mg/kg/200 μL/mouse; *n* = 5) until the 21st day. As another control, normal mice were treated via a feeding needle with tap water (untreated [UTR]; *n* = 5). On days −7, 0, 7, 14, and 21, mice were anesthetized, and blood was taken from the heart. Blood was mixed with 1/10 volume of 3.8% sodium citrate and centrifuged at 3,000 × *g* at 4°C for 10 minutes using a desktop cooling centrifuge. Citrated plasma was stored at −80°C until use. Plasma TAT concentration was measured using a mouse plasma TAT ELISA kit (ab137994; Abcam). The normal range of TAT concentration in BALB/c mouse plasma was 0.4 to 1.3 ng/mL when measured using this kit.

### Ethical Statement for Animal Studies

Experiments with mice were conducted in strict accordance with the recommendations of the Suzuka University of Medical Science Animal Experiment Ethics Committee (approval no. 63), which were prepared according to the guidelines of the Ministry of Education, Culture, Sports, Science, and Technology of Japan. Surgery was performed under pentobarbital anesthesia, and every effort was made to minimize pain.

### Statistical Analysis

Data were collected from five independent experiments and expressed as the mean ± standard deviation (SD). Statistical differences between the different groups were determined using a two-way analysis of variance (ANOVA) followed by Dunnett's or Tukey's multiple comparison test (SPSS version 20; IBM, Armonk, New York, United States). Both Dunnett's and Tukey's tests were used to determine the differences in tumor size of Colon26 cells transplanted into mice and in TAT levels in plasma. Tukey's test was used to compare other data. Statistical significance was considered at *α* = 0.05 ( *p* <0.05) or *α* = 0.01 ( *p* <0.01).

## Results

### Expression of Coagulation and Inflammation Factors in Colon26 Cells

**Fig. 1:**
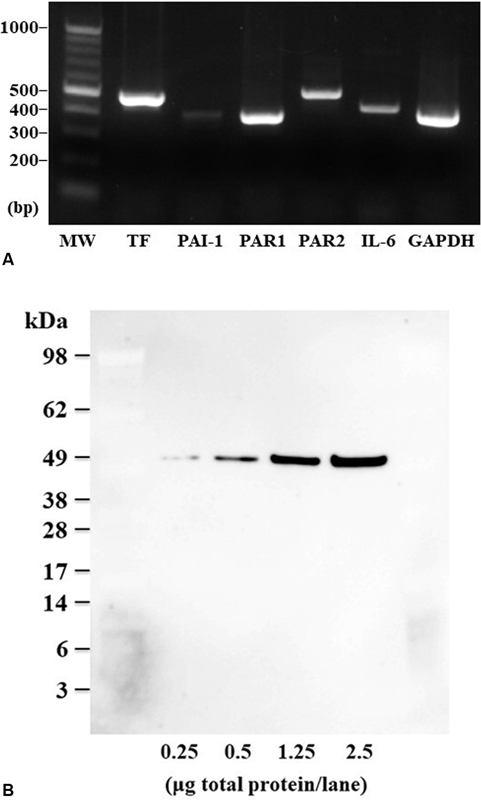
( **A** ) mRNA expression of coagulation and inflammation factor genes in cultured Colon26 cells determined using PCR. GAPDH, glyceraldehyde 3-phosphate dehydrogenase; IL-6, interleukin-6; MW, molecular weight markers; PAI-1, plasminogen activator inhibitor-1; PAR1, protease-activated receptor-1; PAR2, protease-activated receptor-2;; TF, tissue factor. ( **B** ) Western blot image of TF protein in total protein 0.25 to 2.5 μg/lane of a cell extract of cultured Colon26 cells. The data indicate that the molecular weight of TF is approximately 48 kDa.

Although not quantified, Colon26 cells appeared to have high mRNA levels of the TF, PAR1, and PAR2 genes and low mRNA levels of the IL-6 and PAI-1 genes, as shown in Fig. 1A . Coagulation factor VII and factor X could not be detected.

### TF Protein Expression and Activity in Colon26 Cells

As shown in Fig. 1B , Colon26 cells expressed TF protein with a molecular weight of nearly 48 kDa. To measure TF activity in Colon26 cells, the calibration curve of the duration of diluted tissue thromboplastin-induced plasma clotting in the presence of Ca ^2+^ ions was created. As shown in Supplementary Fig. S1 , when the TF activity in Thromborel S was converted to 1 unit/mL, the TF activity from 0.002 unit/mL to 0.05 unit/mL could be sufficiently measured. Using this calibration curve, the TF activity of Colon26 cell suspension was 0.028 ± 0.0015 units/10 ^6^ cells/mL. This corresponds to approximately 2.8% of the TF concentration of Thromborel S.

### Effect of DOACs on Tumor Growth and Tumor-Related Factors in Colon26-Inoculated Mice

**Fig. 2:**
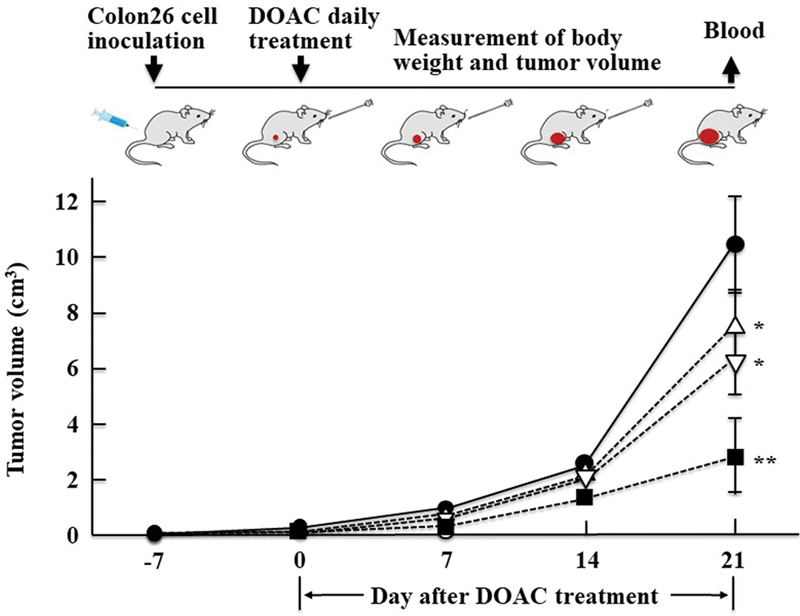
Effect of direct oral anticoagulants (DOACs) on tumor volume in mice inoculated with Colon26 cells. The general experimental procedure is shown at the top of the figure. Mice were inoculated with Colon26 cells, and 7 days later (day 0), DOAC or water was orally administered daily for 21 days. Tumor volumes in Colon26-inoculated mice were measured weekly until day 21; water-treated group ( *n* = 5) (●), dabigatran etexilate (DABE)-treated group ( *n* = 5) (△), rivaroxaban (RVX)-treated group ( *n* = 5) ( **▽** ), edoxaban (EDX)-treated group ( *n* = 5) (▪). Data for tumor volumes on day 21 are shown as mean ± SD. Significant differences in tumor volume compared with that in the water-treated group of Colon26-inoculated mice are shown as * *p* <0.05 and ** *p* <0.01.

Fig. 2 shows the schematic experimental procedure and the effect of each DOAC on tumor volume in Colon26-inoculated mice. No effect on body weight was observed in any of the drug-treated groups of Colon26-inoculated mice compared with untreated (UTR) mice ( Supplementary Fig. S2 ). Tumor volume in Colon26-inoculated mice treated daily with DOAC was significantly reduced compared with that in water-treated Colon26-inoculated mice.

Table 2 shows the tumor volume measured in Colon26-inoculated mice that were orally treated with water or DOAC daily for 21 days. The effects of each DOAC on the tumor volume in Colon26-inoculated mice can be described as follows by considering the tumor volume in water-treated group to be 100%: DABE-treated group: 69% ( *p* <0.05); RVX-treated group: 59% ( *p* <0.05); and EDX-treated group: 28% ( *p* <0.01). The tumor volumes in the EDX-treated group were significantly lower than those in the DABE-treated and RVX-treated groups ( *p* <0.01), indicating that EDX was the most effective inhibitor of Colon26 tumor growth among the tested DOACs.

**Table 2:** Tumor volume and plasma levels of IL-6 and MMP-2 in untreated (UTR) mice and Colon26-inoculated mice orally treated with water or direct oral anticoagulant (DOAC) daily for 21 d

		Colon26-inoculated mice			
	UTR mice	Water	DABE	RVX	EDX
Tumor volume (cm ^3^ )	0	10.6 ± 1.3	7.3 ± 1.7 a	6.3 ± 1.8 a	3.0 ± 1.3 b, c, d
IL-6 (pg/mL plasma)	11.4 ± 3.8	128.4 ± 10.5 e	68.2 ± 7.4 b	75.5 ± 11.3 b	41.6 ± 8.8 b c d
MMP-2 (ng/mL plasma)	100.6 ± 9.8	248.6 ± 31.2 e	196.4 ± 8.9 a	171.4 ± 10.5 b, c	105.8 ± 6.6 b c d

Abbreviations: DABE, dabigatran etexilate; EDX, edoxaban; IL-6, interleukin-6; MMP-2, matrix metalloproteinase-2; RVX, rivaroxaban.

Note: Plasma levels of IL-6 and MMP-2 were determined using factor-specific ELISA.

a Significant difference between the water-treated group and DABE- or RVX-treated group in Colon26-inoculated mice ( *p* <0.05).

b Significant difference between the water-treated group and DABE-, RVX-, or EDX-treated group ( *p* < 0.01).

c Significant difference between the DABE-treated group and RVX- or EDX-treated group ( *p* < 0.01).

d Significant differences between the RVX-treated group and EDX-treated group ( *p* < 0.01).

e Significant difference between UTR mice and the water-treated group in Colon26-inoculated mice ( *p* < 0.01). The data are shown as mean ± standard deviation.

As shown in Table 2 , the IL-6 and MMP-2 levels were significantly increased in water-treated Colon26-inoculated mice compared with those in UTR mice ( *p* <0.01). The effects of each DOAC on IL-6 levels in Colon26-inoculated mice can be described as follows: water-treated group: set to 100%; DABE-treated group: 53% ( *p* <0.01); RVX-treated group: 59% ( *p* <0.01); and EDX-treated group: 32% ( *p* <0.01). The effects of each DOAC on MMP-2 levels in Colon26-inoculated mice can be described as follows: water-treated group: set to 100%; DABE-treated group: 79% ( *p* <0.05); RVX-treated group: 69% ( *p* <0.01); and EDX-treated group: 43% ( *p* <0.01). Both the IL-6 and MMP-2 levels in the EDX-treated group were significantly lower than those in the DABE- and RVX-treated groups ( *p* <0.01), and the MMP-2 level in EDX-treated group decreased to approximately the same level as that in UTR mice. This data suggests that EDX is the most effective Colon26 tumor growth inhibitor among the DOACs tested, in terms of suppressing inflammatory responses and cancer cell invasion. Therefore, our next study was focused on investigating the mechanism underlying the antitumor action of EDX.

### Effects of EDX on Tumor Growth, Tumor Tissue Morphology, and Plasma Levels of Coagulation- and Tumor-Related Factors in Colon26-Inoculated Mice

**Fig. 3:**
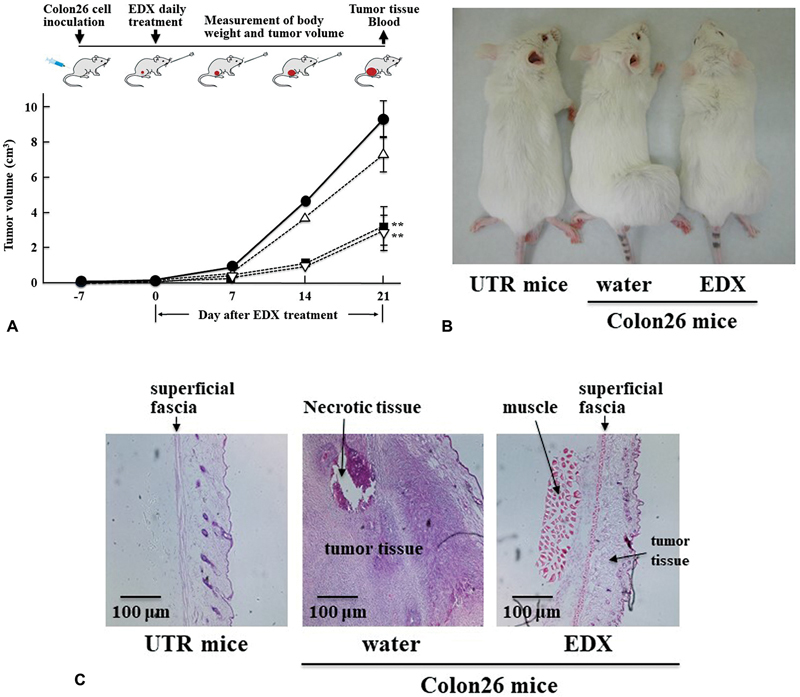
( **A** ) Effect of different concentrations of edoxaban (EDX) on tumor volume in mice inoculated with Colon26 cells. The general experimental procedure is shown at the top of the figure. The thighs of mice were inoculated with Colon26 cells, and 7 days later (day 0), water or EDX (5, 10, or 20 mg/kg/d) was orally administered daily for 21 days; water-treated group ( *n* = 5) (●), EDX (5 mg/kg/d)-treated group ( *n* = 5) (△), EDX (10 mg/kg/d)-treated group ( *n* = 5) (▪), EDX (20 mg/kg/d)-treated group ( *n* = 5) ( **▽** ). The tumor volume was measured once a week until day 21. Day 21 data are shown as mean ± SD. Significant differences in tumor volume compared with that in the water-treated group of Colon26-inoculated mice are shown as ** *p* <0.01. ( **B** ) Images of untreated (UTR) mice and the water-treated group or EDX (10 mg/kg/d)-treated group of Colon26-inoculated mice on day 21. ( **C** ) Hematoxylin-eosin (HE) staining of subcutaneous normal thigh tissue of untreated (UTR) mice and subcutaneous tumor tissue of water-treated group or EDX (10 mg/kg/d)-treated group of Colon26-inoculated mice on day 21. Approximately the same tissue images were observed in each group of individual mice.

Fig. 3A shows a schematic of the experimental procedure and the effect of different concentrations of EDX (5, 10, and 20 mg/kg/d) administered daily for 21 days on the tumor volume in Colon26-inoculated mice. The tumor volume in Colon26-inoculated mice on day 21 can be described as follows by considering the tumor volume in the water-treated group to be 100%: EDX 5 mg/kg/d group: 78%; EDX 10 mg/kg/d group: 34% ( *p* <0.01); and EDX 20 mg/kg/d group: 30% ( *p* <0.01). EDX suppressed tumor growth in a dose-dependent manner, but there was no significant difference between the 10 mg/kg/d and 20 mg/kg/d treatment groups. Thus, 10 mg/kg/d was designated as the appropriate dose for oral EDX treatment, and the following experiments were performed at this dose. Fig. 3B shows representative images depicting a comparison of thigh tumor sizes between water-treated mice and EDX (10 mg/kg/d)-treated mice inoculated with Colon26 cells on day 21. UTR (untreated non-cancer) mice (10 weeks old) were compared as controls. Fig. 3C shows the HE staining morphology of tumor tissues in the thigh subcutaneously inoculated with Colon26 cells and normal thigh tissue of UTR mice. Tumor tissue, which was significantly increased in water-treated Colon26-inoculated mice, was significantly decreased in EDX-treated Colon26-inoculated mice. Most tissues in the water-treated group of Colon26-inoculated mice comprised tumor tissue, and the tissues in the EDX-treated group appeared to be a mixture of normal and tumor tissue.

Table 3 shows the tumor volume and plasma levels of TF, PAI-1, IL-6, and MMP-2 on day 21 in UTR mice and water-treated or EDX-treated Colon26-inoculated mice. All of these plasma factors were significantly increased in water-treated Colon26-inoculated mice compared with their levels in the UTR mouse ( *p* <0.01). These factors, as well as the changes in tumor size, were significantly decreased in the EDX-treated group compared with the water-treated group of Colon26-inoculated mice ( *p* <0.01).

**Table 3:** Tumor volume and plasma levels of coagulation- and tumor-related factors in untreated (UTR) mice and Colon26-inoculated mice orally treated with water or edoxaban (EDX) daily for 21 d

		Colon26-inoculated mice	
	UTR mice	Water	EDX
Tumor volume (cm ^3^ )	0	9.3 ± 1.0 a	3.0 ± 1.0 a, b
TF (pg/mL plasma)	24.2 ± 5.1	75.4 ± 5.3 a	50.7 ± 6.8 a, b
PAI-1 (ng/mL plasma)	17.5 ± 2.4	40.6 ± 3.8 a	33.8 ± 2.1 a, b
IL-6 (pg/mL plasma)	10.2 ± 7.2	130.8 ± 9.4 a	47.2 ± 4.7 a, b
MMP-2 (ng/mL plasma)	101.5 ± 15.8	246.0 ± 39.1 a	106.2 ± 10.0 b

Abbreviations: IL-6, interleukin-6; MMP-2, matrix metalloproteinase-2; PAI-1, plasminogen activator inhibitor-1; TF, tissue factor.

Note: Plasma levels of TF, PAI-1, IL-6, and MMP-2 were determined using factor-specific ELISA.

a Significant difference from the UTR mice group ( *p* <0.01).

b Significant difference from the water-treated group in Colon26-inoculated mice ( *p* <0.01). The data are shown as mean ± standard deviation.

### Effect of EDX on the Expression of Coagulation Factor Receptors, Tumor Cell Transcription, and Growth Factors in Tumor Tissues of Colon26-Inoculated Mice

The effect of EDX on the protein expression of the coagulation factor receptors, PAR1 and PAR2, and of the intracellular factors associated with tumor cell proliferation, STAT3, cyclin D1, and Ki67, in the tumor tissues of Colon26-inoculated mice was examined by immunofluorescence analysis and ELISA.

**Fig. 4:**
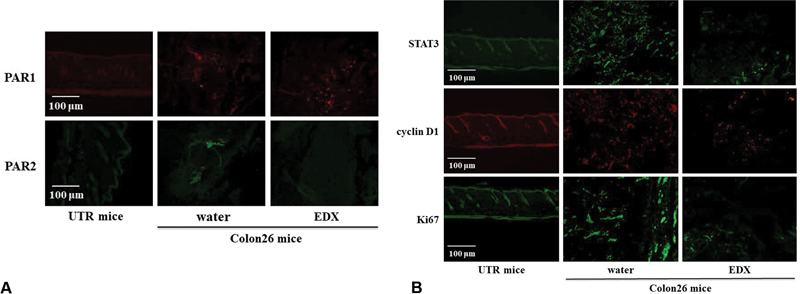
( **A** ) Immunofluorescence data for PAR1 and PAR2 expression in the normal thigh tissues of untreated (UTR) mice and tumor tissues of the water-treated group or edoxaban (EDX) (10 mg/kg/d)-treated group of Colon26-inoculated mice on day 21. ( **B** ) Immunofluorescence data for STAT3, cyclin D1, and Ki67 expression in normal thigh tissues of untreated (UTR) mice and tumor tissues of the water-treated group or EDX-treated group of Colon26-inoculated mice on day 21.

Table 4 shows the tissue levels of coagulation- and tumor-related factors in UTR mice and Colon26-inoculated mice that were orally treated with water or EDX daily for 21 days. Both PAR1 and PAR2 in the tumor tissues of water-treated group mice were significantly increased compared with those in the normal thigh tissue of UTR mice ( *p* <0.01). The PAR1 expression level in the tumor tissues from the EDX-treated group did not change significantly compared with that from the water-treated group, but the PAR2 expression level in the tumor tissues from the EDX-treated group was significantly reduced compared with that from the water-treated group ( *p* <0.01). Fig. 4A shows immunofluorescence data for PAR1 and PAR2 expression levels in normal or tumor tissues. The data show increased levels of PAR1 and PAR2 and decreased levels of PAR2 rather than PAR1 in tumor tissues of EDX-treated mice.

**Table 4:** Tissue levels of coagulation- and tumor-related factors in untreated (UTR) mice and Colon26-inoculated mice orally treated with water or edoxaban (EDX) daily for 21 d

		Colon26-inoculated mice	
	UTR mice	Water	EDX
PAR1 (ng/100 mg tissue)	12.0 ± 4.7	65.2 ± 10.1 a	66.3 ± 9.9 a
PAR2 (ng/100 mg tissue)	18.4 ± 6.5	96.2 ± 16.6 a	20.7 ± 7.3 b
STAT3 (ng/100 mg tissue)	12.5 ± 9.9	157.9 ± 8.4 a	73.9 ± 7.6 a b
cyclin D1 (ng/100 mg tissue)	1.3 ± 0.7	13.1 ± 0.7 a	3.7 ± 0.6 a, b
Ki67 (ng/100 mg tissue)	24.9 ± 5.7	135.0 ± 16.4 a	49.9 ± 5.7 a, b

Abbreviations: PAR1, protease-activated receptor 1; PAR2, protease-activated receptor 2; STAT3, signal transducer and activator of transcription-3.

Note: Tissue levels of PAR1, PAR2, STAT3, cyclin D1, and Ki67 were determined using factor-specific ELISA.

a Significant difference from the UTR mice group ( *p* <0.01).

b Significant difference from the water-treated group in Colon26-inoculated mice ( *p* <0.01). The data are shown as mean ± standard deviation.

Table 4 also shows the concentrations of STAT3, cyclin D1, and Ki67 in the normal tissue of UTR mice and in the tumor tissue of the water-treated group of Colon26-inoculated mice. These levels were all significantly increased in the tumor tissue of the water-treated group compared with those in the normal thigh tissues of UTR mice ( *p* <0.01) and significantly decreased in the tumor tissues of EDX-treated group compared with the water-treated group ( *p* <0.01). Fig. 4B shows the immunofluorescence data showing the expression of STAT3, cyclin D1, and Ki67 in the normal tissues of UTR mice and in the tumor tissues of water- or EDX-treated Colon26-inoculated mice. The expression levels of STAT3, cyclin D1, and Ki67 in the water-treated group of Colon26-inoculated mice were clearly increased compared with those in the normal thigh tissue of UTR mice, while the levels in the tumor tissue of the EDX-treated group were clearly decreased compared with those in the water-treated group.

### Effect of EDX on Apoptosis and p53 Protein Expression in Tumor Tissues of Colon26-Inoculated Mice

**Fig. 5:**
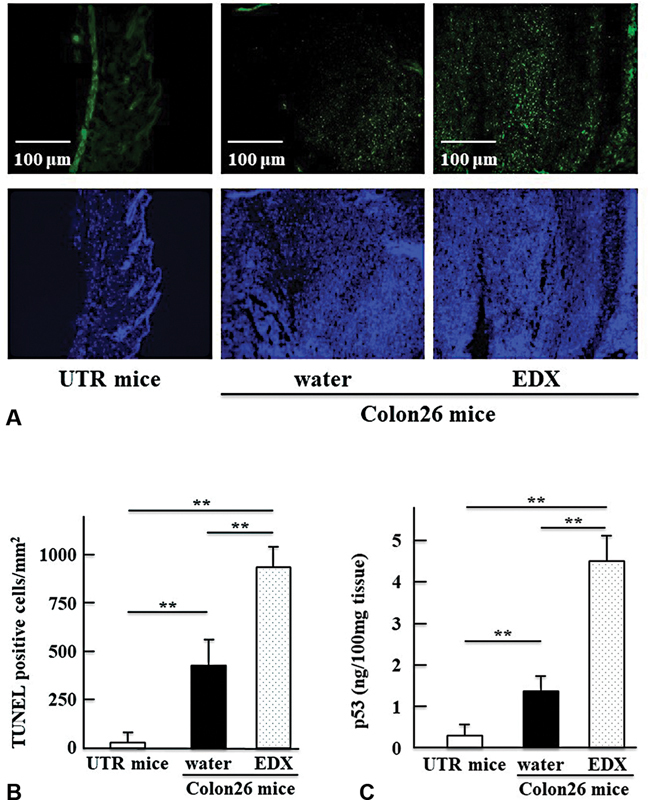
( **A** ) Images of immunofluorescence analysis of TUNEL-positive cells and control DAPI staining in normal thigh tissue of untreated (UTR) mice and tumor tissue in the water-treated group or edoxaban (EDX) (10 mg/kg/d)-treated group of Colon26-inoculated mice on day 21. ( **B** ) TUNEL-positive cells/mm ^2^ in each group of tissues shown in Fig. 5A were counted as described in the text. The data are shown as mean ± SD. Significant differences in TUNEL-positive cells between groups are shown as ** *p* <0.01. ( **C** ) The concentration of p53 protein, determined by ELISA, in solubilized samples (100 mg) of normal thigh tissue from untreated (UTR) mice and tumor tissues from the water-treated group or EDX-treated group of Colon26-inoculated mice on day 21. The data are shown as mean ± SD. Significant differences in p53 protein concentrations between groups are shown as ** *p* <0.01.

Fig. 5A shows TUNEL staining of normal thigh tissue in UTR mice and tumor tissue of water-treated or EDX-treated groups of Colon26-inoculated mice. The tissues were DAPI stained for comparison. As shown in Fig. 5B , TUNEL-positive cells in the tumor tissue of the water-treated group of Colon26-inoculated mice were significantly ( *p* <0.01) higher than those in the normal thigh tissue of UTR mice. In addition, TUNEL-positive cells in the tumor tissue of the EDX-treated group of Colon26-inoculated mice increased significantly ( *p* <0.01) to levels higher than those in the water-treated group. These findings show that EDX administration further increased the number of apoptotic cells in the tumor tissue of mice inoculated with Colon26 cells.

To investigate the possible mechanisms of EDX-associated apoptosis, the levels of p53 protein, which has an antitumor effect by inducing apoptosis in the tumor, 23 were evaluated. Fig. 5C shows the expression levels of p53 protein in normal thigh tissues of UTR mice and tumor tissues of water-treated or EDX-treated group of Colon26-inoculated mice. Levels of p53 protein were significantly ( *p* <0.01) increased in tumor tissue in the water-treated group compared with that in normal thigh tissue and more significantly ( *p* <0.01) increased in tumor tissue in the EDX-treated group. These data show that the expression levels of p53 protein are like those of apoptotic cells in the tumor tissues of water-treated or EDX-treated group of Colon26-inoculated mice, as shown in Fig. 5B .

### Effect of EDX on Angiogenesis in Tumor Tissues of Colon26-Inoculated Mice

**Fig. 6:**
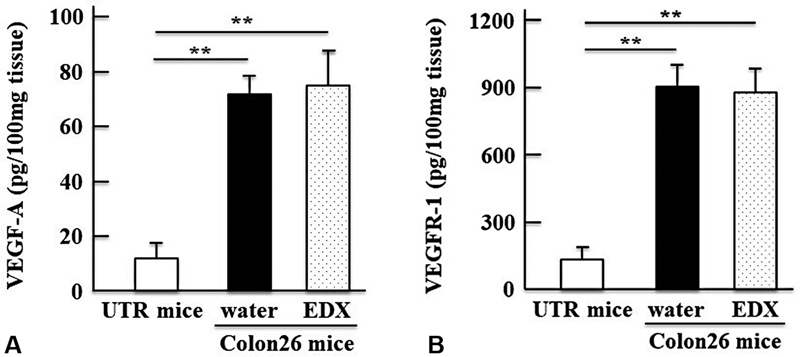
( **A** ) Concentrations of VEGF-A and ( **B** ) VEGFR-1, determined by ELISA, in solubilized samples (100 mg) of normal thigh tissue from untreated (UTR) mice and tumor tissues from water-treated group or EDX-treated group of Colon26-inoculated mice on day 21. The data are shown as mean ± SD. Significant differences in concentrations of the respective proteins between groups are shown as ** *p* <0.01.

As shown in Fig. 6A and B , the expression levels of VEGF-A and VEGFR-1 were significantly ( *p* <0.01) increased in tumor tissues of the water-treated group of Colon26-inoculated mice compared with levels in normal thigh tissue of UTR mice. However, there was no difference in the expression levels of VEGF-A and VEGFR-1 in the tumor tissues between the water-treated and EDX-treated groups of Colon26-inoculated mice. The data shows EDX does not affect tumor angiogenesis.

### Effect of EDX on Coagulation Factor Activation in Colon26-Inoculated Mice

**Fig. 7:**
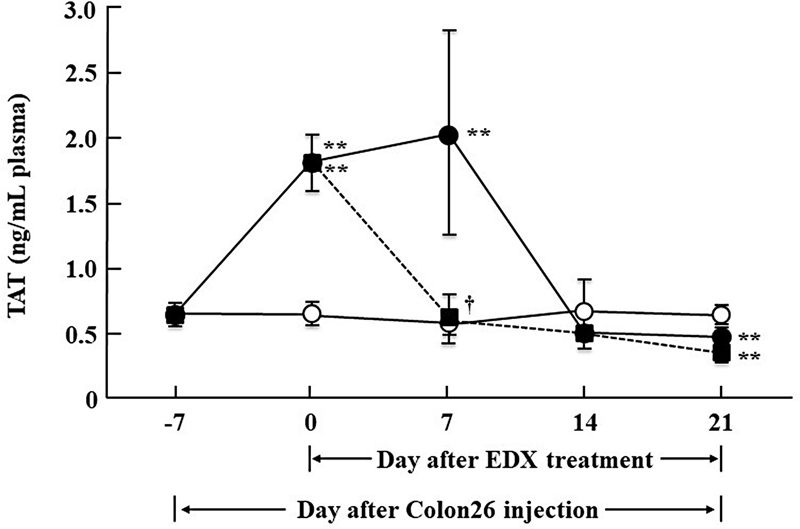
Effect of edoxaban (EDX) on plasma thrombin-antithrombin complex (TAT) levels in untreated (UTR) control mice and Colon26-inoculated mice. Seven days (day 0) after inoculating mice with Colon26 cells, water or EDX (10 mg/kg/d) was orally administered daily for 21 days; citrated plasma samples were prepared from blood drawn from the heart on days -7, 0, 7, 14, and 21 of UTR mice ( *n* = 5) (○), water-treated group ( *n* = 5) (●), or EDX-treated group ( *n* = 5) (▪) of Colon26-inoculated mice. Plasma TAT concentration was determined by ELISA. Significant differences in plasma TAT levels between the water-treated group or EDX-treated group of Colon26-inoculated mice and the UTR mouse group are shown as ** *p* <0.01. Significant differences in plasma TAT levels between the water-treated group and EDX-treated group of Colon26-inoculated mice are shown as ^**†**^ *p* <0.05.

To investigate whether inoculation of Colon26 cells activates blood coagulation in mice and determine how oral administration of EDX affects the coagulation activity, the concentrations of the coagulation marker TAT in the plasma of Colon26-inoculated mice were determined once a week. As shown in Fig. 7 , plasma TAT levels were unchanged in UTR mice (0.6 ± 0.01 ng/mL) but were significantly increased to 1.8 ± 0.2 ng/mL in mice after 7 days of inoculation of Colon26 cells (day 0; *p* <0.001). In the plasma of the water-treated group of Colon26-inoculated mice, TAT levels increased to 2.0 ± 0.7 ng/mL after 2 weeks (day 7) and decreased to the level in UTR mice after 3 weeks (day 14). Conversely, plasma TAT levels in EDX-treated Colon26-inoculated mice from day 0 appeared to decrease to UTR mouse levels within 1 week (day 7) and remained low thereafter. The data show that blood coagulation in mice is activated in the early stages of Colon26 cell inoculation, and this coagulation activation is rapidly suppressed by oral treatment with EDX.

## Discussion

Coagulation factors promote tumor growth; thrombin induces tumor cell proliferation, metastasis, and angiogenesis, 24 enhances tumor cell adhesion and metastatic properties by increasing integrin αIIßIII expression on the tumor cell surface, 25 and induces epithelial-mesenchymal transition via PAR1 in human gastric cancer. 26 In addition, factor Xa alone, factor VIIa-factor Xa complex, 27 and TF-factor VIIa-factor Xa complex 28 are involved in breast cancer cell proliferation via PAR2 activation. Activation of PAR2 is also required for the growth of colon cancer cells 29 and for MMP-2 expression in SW1990 cells. 30 Therefore, coagulation factors have been recognized to play an important role in the growth and metastasis of cancer cells.

This study showed whether mouse colon cancer (Colon26) cells transplanted into a cancer-bearing mouse express coagulation-promoting and pro-inflammatory factors. Colon26 cells expressed TF, PAR1, PAR2, and IL-6 genes as well as TF protein (with a molecular weight of approximately 48 kDa). This TF protein showed considerable coagulation-promoting activity.

The effects of orally administered DABE, RVX, and EDX on the proliferation of Colon26 cells inoculated into BALB/c mice as a syngeneic mouse model were compared. All examined DOACs, especially EDX, significantly reduced the tumor volume by day 21. In addition, the increased plasma IL-6 and MMP-2 levels in Colon26-inoculated mice were significantly decreased in the EDX-treated group compared with those in the DABE-treated and RVX-treated groups.

In our subsequent experiments on the mechanism underlying the antitumor effect of EDX using tumor tissues and blood from the EDX-treated group, histological analysis after HE staining showed that EDX treatment significantly reduced the size of the tumor tissues compared with water treatment. EDX treatment also significantly decreased the plasma levels of TF and PAI-1, both of which have pro-thrombotic and pro-tumorigenic activities, compared with the water-treated group. Furthermore, EDX treatment significantly decreased PAR2 levels, but not PAR1 levels, which were significantly increased in the tumor tissue of water-treated mice.

Subsequent analyses showed that EDX treatment suppressed the expression of STAT3, a transcription factor involved in tumor cell growth, cyclin D1, a cell cycle-related factor, and Ki67, a tumor growth rate marker, which were highly expressed in tumor tissues of Colon26-inoculated mice. These findings indicate that EDX suppresses tumor cell proliferation in mice inoculated with Colon26 cells by suppressing activation of the intracellular signaling system via the factor Xa-PAR2 pathway.

Interestingly, TUNEL staining showed a significant increase in the number of apoptotic cells in the tumor tissue of the water-treated group of Colon26-inoculated mice, whereas EDX treatment further increased the number of apoptotic cells in the tumor tissue of Colon26-inoculated mice. Therefore, the expression of p53 protein in tumor tissues was studied, because it is a transcription factor that regulates cell proliferation and affects DNA repair, cell proliferation arrest, and cell apoptosis. 23 The data showed that p53 expression levels in the absence or presence of EDX agreed with the number of TUNEL-positive cells. These findings suggest that EDX-induced inhibition of the PAR2 pathway upregulates p53 gene expression and induces apoptosis.

It remains unclear how EDX inhibition of the PAR2 pathway increases p53 protein and apoptosis. It has been reported that activation of PAR2 suppresses apoptosis, 31 and suppression of the PAR2 pathway by PAR2 antagonists inhibits STAT3 signaling and causes apoptosis of cervical cancer cells. 32 Meanwhile, thrombin reportedly exerts a bimodal effect on PAR1 in tumor cells by promoting growth at low concentrations while simultaneously impairing growth due to apoptosis at higher concentrations. 33 In addition, thrombin modifies the growth, proliferation, and apoptosis of human colonic organoids via a PAR1- and PAR4-dependent mechanism. 34 These reports may support the hypothesis that factor Xa-PAR2 pathway signals regulate the PAR1 and/or PAR4 pathway, leading to apoptosis, whereas the EDX-induced suppression of the PAR2 pathway could enhance PAR1 and/or PAR4 pathway-dependent apoptosis.

Because intratumoral angiogenesis is closely related to tumor tissue growth, and PAR2 induces angiogenesis and promotes tumor growth, 35 we analyzed the effect of EDX on the expression of VEGF-A and its receptor VEGFR-1, which are primarily involved in angiogenesis, 36 and found that EDX treatment did not affect the expression levels of both factors, suggesting that the factor Xa-PAR2 pathway is not involved in tumor angiogenesis.

Finally, we evaluated the plasma TAT levels in Colon26-inoculated mice to determine whether biologically active TF-expressing Colon26 cells induce blood coagulation and how EDX administration affects the coagulation in Colon26-inoculated mice. The data suggest that the inoculated Colon26 cells activate mouse blood coagulation, and EDX suppresses factor Xa-dependent PAR2 activation of cancer and peri-cancer cells involved in tumor tissue growth. Notably, the TAT levels in mice inoculated with Colon26 cells decreased to the level of those in untreated control mice within nearly 2 weeks, despite the increase in tumor volume at this stage. This phenomenon is clinically similar to that shown in patients with non-metastatic tumors in whom TAT levels are significantly lower than those in patients with metastatic tumors, which are as low as those in healthy subjects. 37

In this study, DABE also showed mild suppression of tumor growth and decreased plasma IL-6 and MMP-2 levels, suggesting that the PAR1 and PAR4 pathways may also be involved in Colon26 cell proliferation and inflammation induced by Colon26 cells to some extent. In addition, the tumor growth inhibitory effect of EDX was significantly higher than RVX. Both inhibitors are expected to suppress the factor Xa-PAR2 pathway; however, it remains unclear why EDX was more effective than RVX. The inhibitory effects of EDX and RVX on factor Xa ( *K _i_* value) are 0.56 nmol/L 38 and 0.4 nmol/L, 39 respectively, and on thrombin generation by prothrombinase complex are *K _i_* = 2.98 nmol/L 40 and IC _50_ = 2.1 nmol/L, 41 respectively. Therefore, the difference in antitumor effects between EDX and RVX may not be attributed to the differences in the direct inhibitory activity against factor Xa.

In the preliminary experiment, cultured Colon26 cells were stimulated with the factor Xa agonist peptide 2-furoyl-LIGRLO-NH _2_ 42 (which activates mouse PAR2), human factor Xa, EDX only, and human factor Xa + EDX, and the mRNA expression levels of mouse genes (thrombomodulin, MMP9, VEGF, platelet-derived growth factor-α, transforming growth factor-β, and macrophage cell-stimulating factor, besides TF, PAI-1, PAR1, PAR2, and IL-6 shown in Fig. 1A ) were semi-quantitatively determined. The mRNA expression of these genes could be detected, but the levels remained the same regardless of the conditions (data not shown). Therefore, based on the preliminary results, further study is needed to show the effect of EDX on cells in the tumor microenvironment 43 that modulates the progression of cancer cells, such as tumor-associated macrophages producing several proteases, growth factors, cytokines, and cancer-associated fibroblasts producing extracellular matrix.

**Fig. 8:**
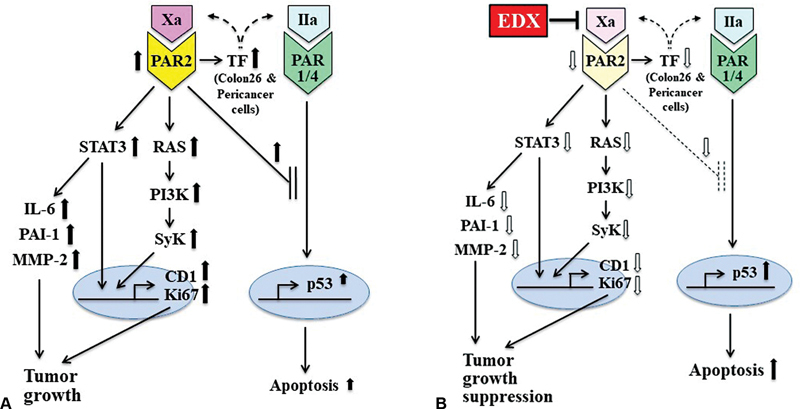
The proposed basic mechanism of the effect of edoxaban (EDX) on tumor growth and apoptosis mediated by factor Xa-PAR2. ( **A** ) In the absence of EDX, tissue factor (TF) expressed in inoculated Colon26 cells activates blood coagulation and inflammation in host mice, and generated factor Xa activates PAR2, whereas generated thrombin possibly activates PAR1/PAR4 on Colon26 cells and various stroma cells in the tumor microenvironment. PAR2 activation stimulates TF expression, STAT3-mediated IL-6, PAI-1, and MMP-2 production in peri-cancer cells, and RAS/PI3K/SyK/Ki67/cyclin D1 signaling pathways in tumor cells ( *black upward arrows* ). These affect tumor growth. In addition, thrombin-mediated PAR1 and PAR4 pathways induce p53 expression, leading to apoptosis, which may be regulated by the PAR2-dependent system. ( **B** ) EDX administration to mice suppresses factor Xa-mediated PAR2 activation, reducing PAR2 levels, followed by a decrease in plasma TF, IL-6, PAI-1, and MMP-2 levels and RAS/PI3K/SyK/Ki67/cyclin D1 signaling pathways ( *white downward arrows* ), leading to tumor growth suppression. Furthermore, suppression of PAR2 pathway activation by EDX may reduce the regulatory system against the PAR1/PAR4 pathway and may increase the p53-dependent apoptosis ( *black upward arrows* ).

Fig. 8 summarizes the presumed basic mechanisms of the inhibitory effect of EDX administration on the growth of Colon26 tumors. In the absence of EDX, as shown in Fig. 8A , TF expressed in inoculated Colon26 cells activates blood coagulation and inflammation in host mice, and generated factor Xa activates PAR2, whereas thrombin possibly activates PAR1/PAR4 on Colon26 cells and various stroma cells in the tumor microenvironment. Activation of these cells produces pro-thrombotic and pro-tumorigenic TF and activates STAT3-mediated IL-6, PAI-1, and MMP-2 production (black upward arrows), and RAS/PI3K/SyK/Ki67/cyclin D1 signaling pathways, leading to tumor growth. In addition, the thrombin generated may activate the PAR1 and PAR4 pathways that induce p53 expression, leading to apoptosis, which may be regulated by the PAR2-dependent system. Under these conditions, as shown in Fig. 8B , inhibition of factor Xa by EDX administration to mice suppresses PAR2 activation, reducing PAR2 levels, followed by a downregulation of the PAR2-mediated STAT3/IL-6 signaling pathway and the RAS/PI3K/SyK/Ki67/cyclin D1 signaling pathways (white downward arrows), resulting in tumor growth suppression.

Furthermore, although the regulatory mechanism of the PAR1/PAR4 pathway by the PAR2 pathway is unclear, suppression of PAR2 pathway activation by EDX may reduce the regulatory system against the PAR1/PAR4 pathway and increase the p53-dependent apoptosis (black upward arrows). The detailed mechanism underlying apoptosis induction by EDX administration remains to be confirmed in further studies soon using PAR1 and PAR4 knockout mice.

While the current study was in progress, Buijs et al 44 reported the effects of DABE and RVX on tumor growth and metastasis in xenograft models in which human breast cancer MDA-MB-231 and human triple-negative breast cancer cells were transplanted into immunodeficient NOD/SCID/γc −/− (NSC) mice; the authors concluded neither of the DOACs had antitumor effects. In addition, Najidh et al 45 summarized in a systematic review the effects of DOACs on cancer growth and metastasis in animal models, in which the authors concluded that DOAC monotherapy showed a lack of effect in mouse xenograft models and that the effect on cancer growth and metastasis in mouse syngeneic models depended on the timing of DOAC treatment and type of cancer model used. Given the current results using a syngeneic mouse model and the above results, certain DOACs may suppress the growth and metastasis of certain cancers in patients receiving a daily dose of DOACs to prevent the development of thrombosis.

This study has a few limitations. First, the number of mice in each group was small. Although we conducted many experiments and the data obtained are reliable, we used the minimum number of animals necessary for maintaining animal welfare and shortening the analysis time. However, it is believed that including larger number of animals per group will give more accurate and clearer statistical results. Second, to determine the antitumor effect of DOACs, we mainly used ELISA and immunofluorescence analysis for evaluating the changes in the factors in tumor tissues. However, more detailed results can be obtained by analyzing changes in gene expression using real-time PCR and microarray analysis, which we intend to do in future studies. Third, to analyze the effect of EDX on angiogenesis, we investigated the VEGF-A and VEGFR-1 expression and concluded that the factor Xa-PAR2 pathway was not involved in the antitumor effects of EDX administration. However, many factors other than VEGFA are involved in tumor angiogenesis, including basic fibroblast growth factor, angiopoietin, hepatocyte growth factor, epidermal growth factor, and placental-derived growth factor. 36 Therefore, these factors should also be analyzed in the future. Lastly, as the experiments in this study were performed on mice, it is unclear whether the antitumor effect of EDX observed in these mice can be observed in humans to the same extent. Therefore, it is necessary to prospectively analyze whether the antitumor effects of DOACs are clinically observed in thrombosis patients treated with DOACs.

## Notes

**What Is Known about This Topic?**

- Some anticoagulants inhibit tumor growth in mouse models.
- The effect of direct oral anticoagulants (DOACs) on cancer growth and metastasis in mouse syngeneic models depends on the timing of DOAC treatment and type of cancer model used.

What Does This Paper Add?

- The factor Xa-specific DOAC edoxaban (EDX) significantly suppressed the growth of Colon26 tumor cells inoculated into BALB/c mice.
- EDX suppressed elevated plasma levels of IL-6, MMP-2, TF, and PAI-1 and elevated tumor tissue levels of PAR2, STAT3, cyclin D1, and Ki67 in Colon26-inoculated mice.
- EDX suppressed the factor Xa-PAR2 pathway and tumor cell proliferation and induced tumor cell apoptosis in a p53-dependent manner.
