# Supplementary material for: Edoxaban, a Factor Xa-Specific Direct Oral Anticoagulant, Significantly Suppresses Tumor Growth in Colorectal Cancer Colon26-Inoculated BALB/c Mice
Source: TH Open. 2023 Jan 7;7(1):e1–e13. doi: 10.1055/s-0042-1758855 (PMC9825203; doi:10.1055/s-0042-1758855)
Supplement: Supplementary file 1 — Supplementary Material [file 10-1055-s-0042-1758855-s22060030.pdf]

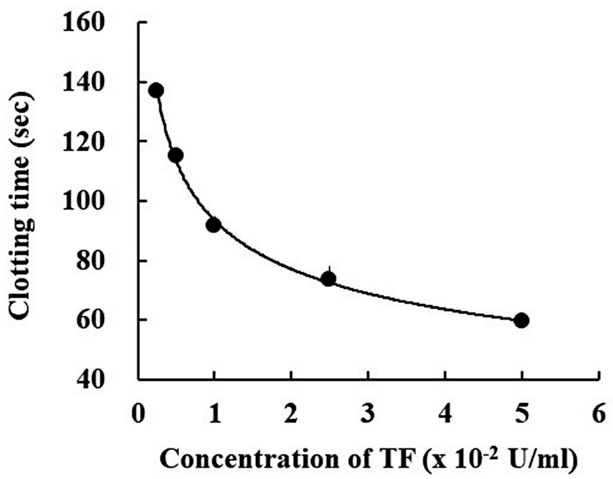

Supplementary Fig. S1 Calibration curve for TF activity measurement in a suspension containing various numbers of Colon26 cells. TF activity was evaluated by measuring the duration of human plasma clotting induced by tissue thromboplastin in the presence of Ca^{2+} ions. Thromborel S, a tissue thromboplastin reagent, was diluted with Tris-buffered saline from 1/10 to 1/1,000. The TF activity of Thromborel S was converted to 1 unit/mL so that the TF activity from 0.002 unit/mL to 0.05 unit/mL could be adequately measured. The method is described in detail in the text.

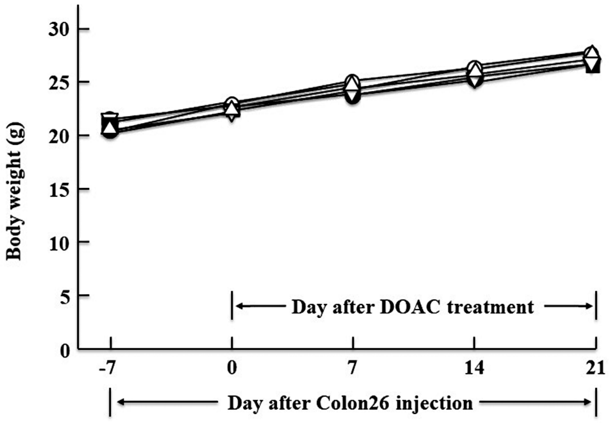

Supplementary Fig. S2 Changes in body weight in each group of untreated (UTR) mice and Colon26-inoculated mice that were orally administered DOACs. UTR mice ($n = 5$) (○), water-treated group ($n = 5$) (●), dabigatran etexilate (DABE)-treated group ($n = 5$) (△), rivaroxaban (RVX)-treated group ($n = 5$) (▽), edoxaban (EDX)-treated group ($n = 5$) (■).

 © 2023. The Author(s).
